# A Multidomain Lifestyle Intervention in Dutch Older Adults to Prevent Cognitive Decline (FINGER‐NL): from RCT to follow‐up study

**DOI:** 10.1002/alz70860_104896

**Published:** 2025-12-23

**Authors:** Marissa D. Zwan, Kay Deckers, Lisa Waterink, Lion M. Soons, Sonja Beers, Sofie van Houdt, Berrit Stienstra, Judith Z. Kwant, Sophie C.P.M. Wimmers, Rachel A.M. Heutz, Jurgen A.H.R. Claassen, Joukje M. Oosterman, Rianne A.A. de Heus, Ondine van de Rest, Yannick Vermeiren, Richard C Oude Voshaar, Nynke Smidt, Laus M. Broersen, Sietske A.M Sikkes, Esther Aarts, Wiesje M. van der Flier, Sebastian Köhler, MOCIA consortium, FINGER‐NL consortium

**Affiliations:** ^1^ Alzheimer Center Amsterdam, Neurology, Vrije Universiteit Amsterdam, Amsterdam UMC location VUmc, Amsterdam, Netherlands; ^2^ Amsterdam Neuroscience, Neurodegeneration, Amsterdam, Netherlands; ^3^ Department of Psychiatry and Neuropsychology, Alzheimer Centrum Limburg, Mental Health and Neuroscience Research Institute (MHeNs), Maastricht University, Maastricht, Netherlands; ^4^ Division of Human Nutrition and Health, Wageningen University & Research, Wageningen, Netherlands; ^5^ Division of Human Nutrition and Health, Wageningen University & Research, Wageningen, The Netherlands., Wageningen, Netherlands; ^6^ HAN University of Applied Sciences, Nijmegen, Netherlands; ^7^ Department of Epidemiology, University of Groningen, University Medical Center Groningen, Groningen, Netherlands; ^8^ Department of Epidemiology, University of Groningen, University Medical Center Groningen, Groningen, The Netherlands., Groningen, Netherlands; ^9^ Department of Geriatrics, Radboud University Medical Center, Radboudumc Alzheimer Center, Nijmegen, Netherlands; ^10^ Department of Geriatrics & Radboudumc Alzheimer Center, Radboud University Medical Center, Nijmegen, Netherlands, Netherlands; ^11^ Radboud University, Donders Institute for Brain, Cognition and Behaviour, Nijmegen, Netherlands; ^12^ Department of Cardiovascular Sciences, University of Leicester, Leicester, United Kingdom; ^13^ Department of Geriatrics & Radboudumc Alzheimer Center, Radboud University Medical Center, Nijmegen, Netherlands; ^14^ Department of Primary and Community Care, Radboud University Medical Center, Nijmegen, Netherlands; ^15^ Department of Psychiatry, University of Groningen, University Medical Center Groningen, Groningen, Netherlands; ^16^ Danone Research & Innovation, Utrecht, Netherlands; ^17^ Department of Clinical, Neuro and Developmental Psychology, VU University, Amsterdam, Netherlands; ^18^ Epidemiology and Data Science, Vrije Universiteit Amsterdam, Amsterdam UMC location VUmc, Amsterdam, Noord‐Holland, Netherlands; ^19^ Alzheimer Center Amsterdam, Neurology, Amsterdam UMC Location VUmc, Vrije Universiteit Amsterdam, Amsterdam, Netherlands

## Abstract

**Background:**

FINGER‐NL aims to investigate the effectiveness of a 2‐year multidomain lifestyle intervention to maintain optimal cognitive functioning in Dutch at‐risk older adults. Here we provide an update on the progress and follow‐up of this study.

**Methods:**

Embedded in the World‐Wide FINGERS initiative and as part of the MOCIA research programme, FINGER‐NL is a 24‐month Dutch multi‐center, randomized, controlled, multidomain lifestyle intervention trial among adults at risk for cognitive decline. Inclusion criteria are 60‐80 years of age and presence of both modifiable (based on LIfestyle for BRAin health (LIBRA) score) and non‐modifiable (positive family history of dementia or self‐reported subjective cognitive decline) risk factors. Participants were randomized to either a high‐intensity (personalized, supervised hybrid program with group meetings and individual sessions) or a low‐intensity (digital information) lifestyle intervention group. Efforts are ongoing to expand and enrich FINGER‐NL

**Results:**

Between February 2022 and May 2023, we screened 2057 individuals and randomly assigned 1210 eligible individuals to the high‐intensity (*n* = 605) or low‐intensity group (*n* = 605). One‐year follow‐up assessments have been completed and carried out in 544 (90%) participants in the high‐intensity group and 505 (83%) in the low‐intensity group. Two‐year follow‐up assessments are ongoing, with currently 369 completed assessments in the high‐intensity group (and *n* = 52 (12%) participants lost to follow‐up) and 358 completed assessments in the low‐intensity group (and *n* = 92 (20%) participants lost to follow‐up). Using stored biomaterials, blood‐based biomarker analyses and measurement of genetic variations are planned in 2025. Funding has been secured for additional follow‐up measurements at three and four years after baseline to determine sustained effects on cognitive performance and lifestyle after completion of the intervention. Currently, 456 (74%) participants who completed the intervention consented to participate in the extension study.

**Conclusion:**

FINGER‐NL is progressing well. Point of attention is participant adherence, especially in the low‐intensity group. There is high interest among participants to continue their participation with additional follow‐up measurements after completion of the intervention. Extension of the project and (inter) national (joint) initiatives expand and enrich FINGER‐NL, which enables and stimulates data sharing and joint analyses across WW‐FINGER studies.

